# Management of Constipation in Hospitalized Patients

**DOI:** 10.3390/jcm12196148

**Published:** 2023-09-23

**Authors:** Gregory S. Sayuk, Qi T. Yu, Corey Shy

**Affiliations:** 1 Division of Gastroenterology, Washington University School of Medicine, St. Louis, MO 63110, USA; 2 Hospice and Palliative Medicine, Loma Linda University Health, Loma Linda, CA 92354, USA; 3 Division of Hospital Medicine, Department of Medicine, Barnes-Jewish Hospital, Washington University School of Medicine, St. Louis, MO 63110, USA

**Keywords:** constipation, opioid-induced constipation, inpatient, laxative, treatment

## Abstract

Constipation is frequently encountered in hospital settings and can have potentially serious consequences yet is often underrecognized and undertreated. Opioid-induced constipation is a common cause of constipation in hospitalized patients. Opioids induce constipation through agonistic effects on enteric µ-opioid receptors. This review aims to provide insight on the identification and management of constipation in inpatient settings, with a particular focus on opioid-induced constipation. Constipation assessment should be routinely initiated at hospital admission and can be facilitated by thorough symptom assessments; relevant patient history, including recent medication use; physical examination; and patient assessment tools developed to evaluate the impact of constipation. Management of opioid-induced constipation should begin with ensuring adequate hydration and electrolyte balance and encouraging patient mobilization. Other treatments may include laxatives, enemas, intestinal secretagogues, peripherally acting µ-opioid receptor antagonists, and manual disimpaction. Surgical intervention may be required for some patients as a salvage therapy in severe, refractory cases.

## 1. Introduction

**Figure 1:**
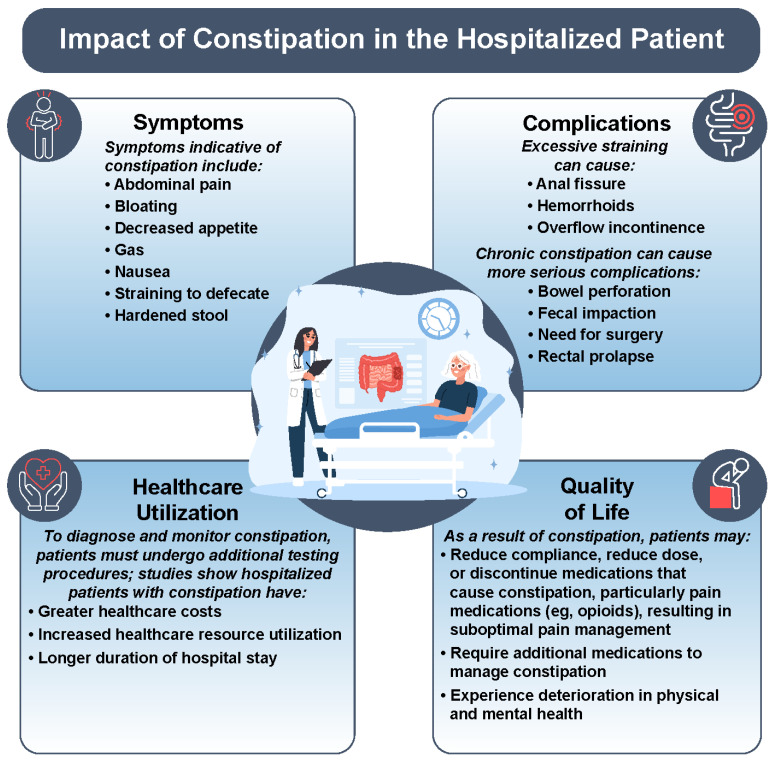
Impact of constipation in the hospitalized patient [2,7,10,11,12,13,14].

Constipation is a debilitating condition characterized by infrequent, hard, and difficult-to-pass stools and is often associated with gastrointestinal symptoms, including abdominal pain, flatulence, bloating, nausea, and decreased appetite (Figure 1) [1,2]. Constipation occurs frequently, affecting approximately 33 million adults in the United States and resulting in 92,000 hospitalizations each year [3]. Elderly individuals are particularly prone to constipation, with a prevalence around 30% in those aged >70 years [4].

Constipation may be the primary indication for hospital admission or might develop during hospital stay (“hospital-acquired”) [1]. Hospital admission can exacerbate existing constipation and increase the risk for development of new constipation due to multiple factors, including immobility, dehydration, an altered diet, and the prescription of certain medications, such as opioids [5]. A common side effect of opioids is constipation. Opioid-induced constipation (OIC) is especially prevalent in hospital settings and requires increased awareness and consideration [6].

Wide variations in the reported incidence of constipation in hospitalized patients (5% to 90.5%) can be attributed in part to a lack of a standard definition for constipation in the hospital setting [7]. The Rome IV criteria are widely used to define constipation [8]. However, this definition is intended to identify chronic constipation in outpatient settings and is not intended to be applied in hospitalized settings, where symptoms may be more acute and multifactorial in etiology. Simplified definitions of constipation in hospitalized patients include a decreased frequency of bowel movements (BMs) during hospitalization versus preadmission, extended times between BMs (e.g., <3 per week), and/or stools that are excessively hard and difficult to pass [9].

### 1.1. Impact of Constipation in Inpatient Settings

Constipation is associated with many potential consequences (Figure 1) [2,7]. These may result in the increased use of medical services, increased cost of care, and reduced compliance with pain medication regimens (e.g., opioids) (Figure 1) [7,10]. Consequently, patients with constipation may suffer from a significantly impaired quality of life, both in physical and mental health [11]. Furthermore, hospitalized patients with constipation are more likely to have extended hospital stays, increased intensive care unit mortality, and greater difficulty weaning off mechanical ventilation [12,13].

### 1.2. Inpatient Constipation Is Underdiagnosed and Undertreated

Despite the impact on patient symptoms, potential complications, and healthcare utilization, constipation is often unrecognized, especially in hospital settings where patients may present with other acute conditions demanding immediate attention [7]. Constipation symptoms can be especially challenging to detect in the elderly, institutionalized patients, patients on sedatives, and those who are mechanically ventilated, as they may be less communicative and/or aware of constipation symptoms [7,9,15]. A lack of gastrointestinal specialist involvement in managing constipation in hospitalized patients may also contribute to its underrecognition and increase its associated morbidity [12]. Therefore, it is essential to recognize patients who either have or are at risk for developing constipation during their hospital stay. Though there is a degree of overlap in ambulatory and inpatient constipation strategies, this review primarily seeks to provide guidance on the recognition and management of constipation in hospitalized patients, with a focus on OIC.

## 2. Etiology and Presentation

**Figure 2:**
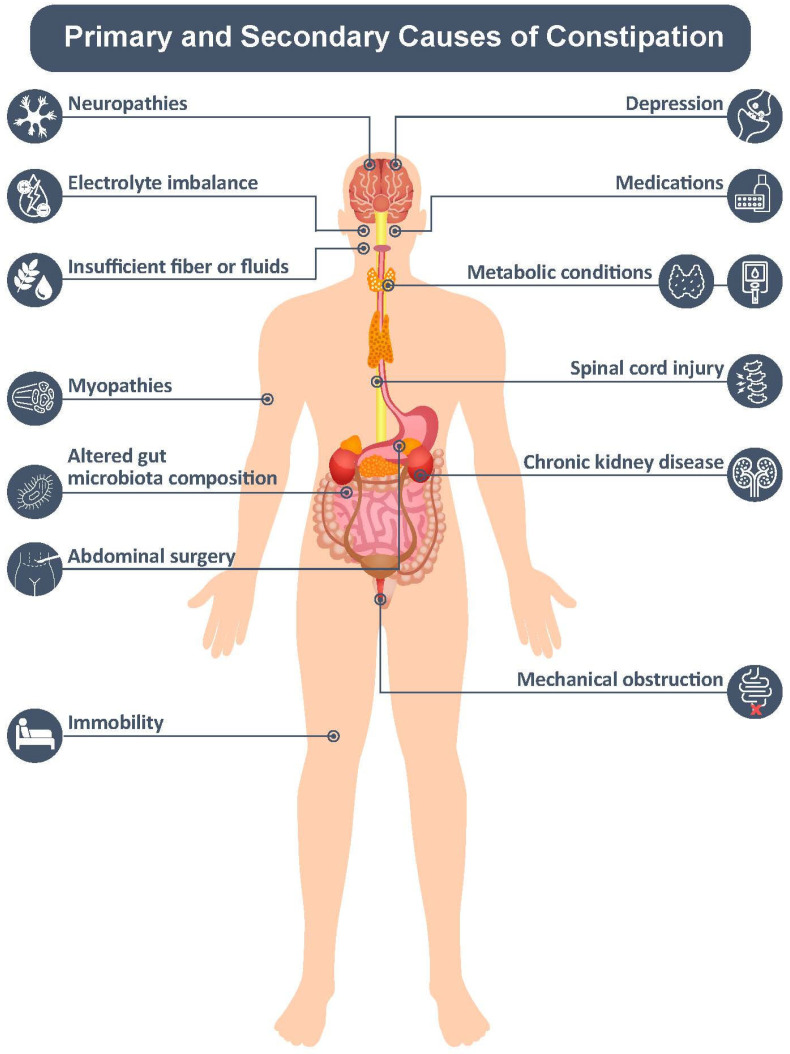
Primary and secondary causes of constipation [7,11,16,17].

Constipation is classified as primary or secondary according to its underlying causes (Figure 2) [16].

### 2.1. Primary Constipation

Primary constipation (idiopathic) can be classified based on symptoms into categories of functional constipation, constipation-predominant irritable bowel syndrome, or unspecified functional bowel disorders [8]. Three subtypes have been defined based on colonic transit: normal transit constipation, slow transit constipation, and disorders of defecation (also known as outlet dysfunction) [11]. Normal transit constipation, the most common subtype, is generally characterized by hard stool consistency but with less profound delays in intestinal transit [3]. Slow transit constipation is characterized by a more delayed passage of feces through the bowel [16]. Outlet dysfunction constipation includes impaired rectal evacuation due to abnormal muscle contraction of the pelvic floor; pelvic floor dyssynergia is a subtype of outlet dysfunction [16]. Many patients experience dyssynergic defecation and slow transit constipation simultaneously [3].

### 2.2. Secondary Constipation

Secondary constipation can occur as a result of drugs and medications (e.g., opioids, antihypertensives, and antidepressants), dietary/lifestyle factors, alterations to the gut microbiota, psychosocial influences, and medical conditions (Figure 2) [7,11,16,17]. Studies have shown that cannabinoids delay gastric emptying and impair colonic motility and tone. However, further studies in humans are needed to determine the effect of cannabinoids on constipation [18].

#### Opioid-Induced Constipation

Opioids exert their effects by binding to μ-opioid receptors, which are highly expressed in both the central and peripheral nervous systems, and to a lesser in the gastrointestinal tract [19,20]. Activation of μ-opioid receptors in the bowel results in decreased intestinal contractility and transit, as well as diminished fluid volume in the gastrointestinal tract [19,21]. Thus, while opioids are often necessary to achieve pain control not possible with other analgesics, their use may be accompanied by numerous undesirable gastrointestinal effects, collectively termed opioid-induced bowel dysfunction, which include constipation-like symptoms. Opioids can also worsen existing constipation. OIC is one of the most common and bothersome symptoms of opioid-induced bowel dysfunction, with prevalence estimates ranging from 40% to 95% of patients on an opioid regimen [22].

Although many opioid-induced side effects, such as nausea and vomiting, typically subside over time, a subset of patients with OIC do not develop tolerance to constipating effects with longer opioid use [23,24]. Consequentially, patients may resort to dose reduction or discontinuation of their opioid regimen, thus compromising their pain management plan [25,26].

## 3. Evaluation and Differential Diagnosis

Considering the multifactorial etiology of constipation in hospitalized patients [7], identifying the cause can be challenging. Screening for symptoms should begin at hospital admission, independent of the patient’s primary complaint [7]. It has been suggested that a formal constipation assessment protocol using stool charts may facilitate the recognition and diagnosis of constipation in inpatient settings [27]. While healthcare providers and patients may feel reluctant to initiate conversations about constipation, early recognition is key to the successful management of constipation in the hospital setting [28].

### 3.1. Initial Evaluation

**Figure 3:**
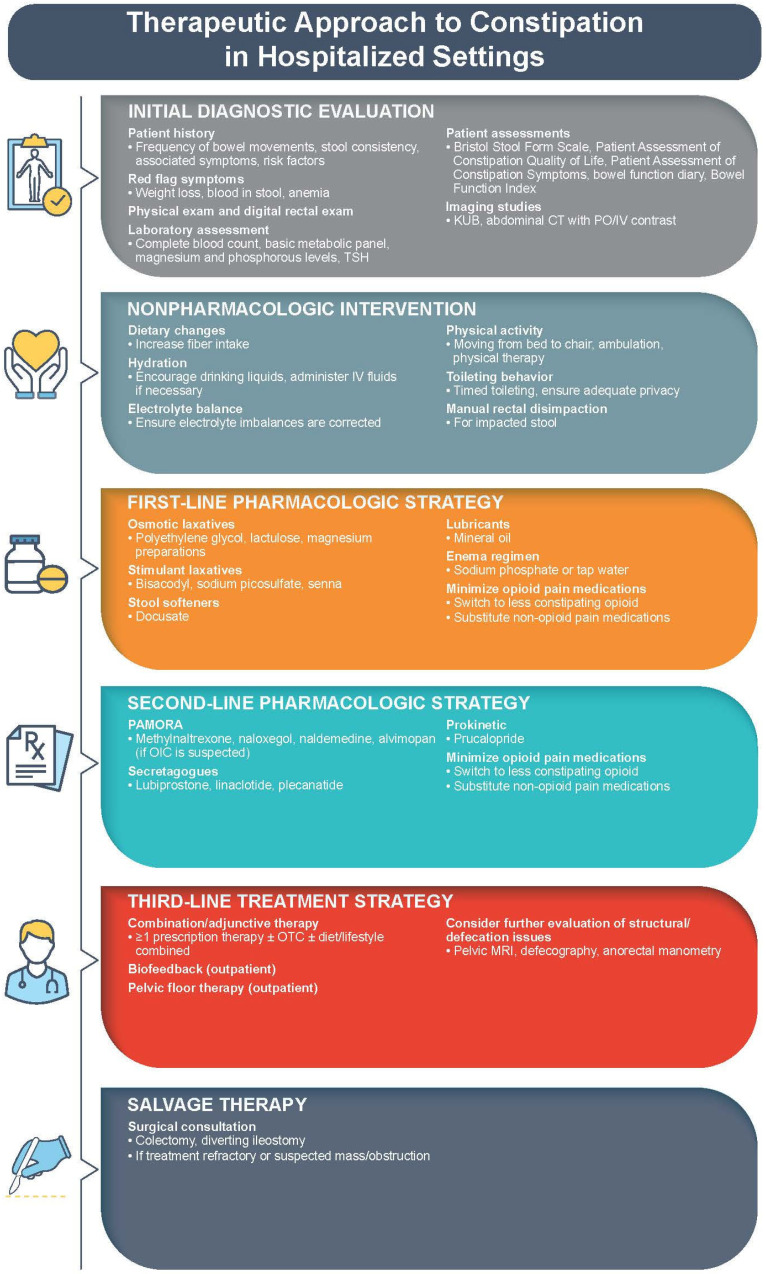
Therapeutic approach to constipation in hospitalized settings [2,7,11,16,23,29,30,31,32,33,34,35,36]. CT: computed tomography; IV: intravenous; KUB: kidney, ureter, and bladder X-ray; MRI: magnetic resonance imaging; OIC: opioid-induced constipation; PO: positive oral; TSH: thyroid-stimulating hormone.

Evaluation should include a thorough symptom assessment, a review of patient history, a medication list, and a physical examination (Figure 3) [7,16,23,24]. Patients should be asked about their normal BM routine, the frequency of feeling the need to defecate [29], the time and consistency of their last stool, whether they are taking laxatives or have a diagnosed bowel disorder [7], and what their dietary habits are with regard to fluid and fiber intake [11,16]. Risk factors should be recognized, including medications, prolonged immobilization, and certain medical conditions (Figure 2) [7,16]. Any concerning symptoms (Figure 3) should prompt pursuit of further diagnostic investigation, including colonoscopy and/or imaging studies [16,30].

Comprehensive physical and neurological examinations can reveal underlying diseases that may cause constipation [16,30]. Suggestive physical examination findings include abdominal distension, decreased or increased bowel sounds (from partial obstruction), and a left quadrant “mass” from retained stool or fecaliths [2]. Inspection of the perineum may reveal hemorrhoids, fissures, rectal prolapse, and skin tags as sequelae or factors exacerbating constipation. The “anal wink” reflex can be elicited using a cotton-tipped applicator in all four quadrants around the anus. The absence of “anal wink” contraction may indicate sacral nerve pathology [29].

A complete digital rectal examination should be performed in patients who do not have contraindications (e.g., severely neutropenic patients, prostatic abscesses, or prostatitis) [2]. Digital rectal examinations provide information on the resting sphincter tone, a potential rectal mass, anal fissures, and impacted stool [2,31]. Detailed rectal examination can also help identify pelvic floor dyssynergia/outlet constipation [30,31].

Following a review of patient history and physical examination, basic laboratory testing may be necessary to identify secondary causes of constipation, including complete blood count (evidence of anemia, raising concern for colorectal malignancy), biochemical profile (assessing for metabolic or electrolyte derangements), serum calcium (hypercalcemia), and thyroid function tests (hypothyroidism) (Figure 3) [16,30].

In addition to physical examination, several patient-assessment tools are available to evaluate the impact of constipation (Figure 3) [23]. For example, the Bristol Stool Form Scale is a visual aid that can help identify stool consistency or form suggestive of constipation. Harder Bristol stool forms (Type 1 and 2) correlate with slower colonic transit time [30].

### 3.2. Imaging

Imaging is an especially important tool for the evaluation of constipation that is quick, widely available, and offers a spectrum of anatomical and functional insights. Abdominal radiography (abdominal plain film or X-ray) is a readily available, inexpensive initial imaging assessment for constipation. Supine anteroposterior images of the abdomen and pelvis (kidney, ureter, and bladder X-ray) can help assess the burden of feces, colon diameter, and evidence of colonic obstruction. Upright and lateral decubitus images can help assess for complications of constipation (e.g., perforation-associated intraperitoneal air). Abdominal plain films are limited in their utility to delineate primary and secondary constipation from other etiologies of bowel distention and stool retention, such as adynamic ileus and intestinal pseudo-obstruction [32].

Computed tomography (CT) scanning is an important imaging modality for assessing constipation, with multiple advantages, including availability, performance ease, extra-colonic structures’ visualization, and superior sensitivity and specificity [32]. CT offers key advantages over plain film imaging, namely greater detail in three dimensions (cross-sectional, axial, and coronal), while still allowing rapid acquisition. Multi-detector CT scanning is especially useful in hospital settings, as it provides faster imaging [32]. CT scans can detect colonic and rectal obstruction, and expose complications of constipation that require immediate attention, such as stercoral ulceration, ischemia, and perforation. As a constipation assessment, abdominal CT ideally should be performed with intravenous or positive oral contrast to optimize evaluation of the bowel wall for masses, ischemia, or inflammation. CT can help detect fecal impaction, bowel obstruction, and/or if there are associated complications, thereby informing management decisions. CT is particularly useful in detecting mechanical causes of constipation [32]. One notable drawback of CT is the considerable radiation exposure required for scans, which may limit its repeated use.

## 4. Differential Diagnosis of Constipation in Hospitalized Patients

### 4.1. Primary Constipation

In patients suspected to have primary constipation, additional diagnostic evaluation can elucidate the subtype. Tests, such as anorectal manometry, the balloon expulsion test, and evaluation of colonic transit with radiopaque markers or wireless motility capsules, are generally performed in the outpatient setting and can be guided by gastroenterology consultation [16,30].

### 4.2. Secondary Constipation

#### 4.2.1. Opioid-Induced Constipation

Diagnosing OIC in the inpatient setting can be challenging, as its symptoms often overlap with other primary/secondary constipation types [28]. In 2014, an expert working group proposed the following OIC definition: a change from baseline in bowel habits when initiating opioid therapy characterized by reduced BM frequency, the development or worsening of straining to pass BMs, a sense of incomplete rectal evacuation, and/or harder stool consistency [28]. Rome IV diagnostic criteria for OIC specify a new or worsening of constipation symptoms following initiation of, changes to, or increases in opioid therapy, as manifested by ≥2 of the following during ≥25% of BMs: lumpy or hard stools (i.e., Bristol Stool Form Scale Type 1 or 2), straining, sensation of incomplete evacuation, sensation of blockage, use of manual maneuvers (digital manipulation/pelvic floor support), and/or <3 spontaneous BMs per week [8]. As alleviation of pain may be a primary concern of healthcare providers, OIC may be underrecognized [28].

#### 4.2.2. Ileus

Ileus manifests as bowel distension, a lack of bowel sounds, a buildup of gas/fluids, and delayed stool evacuation [19,37]. Symptoms include nausea, vomiting, and stomach cramps. Postoperative ileus occurs after surgical manipulation in abdominal surgery patients and its pathophysiology involves a complex interplay between the surgical stress response, the inflammatory response, and the effect of exogenous and endogenous opioids [19]. Acute illness, immobility, and electrolyte derangements can also contribute to ileus [38].

#### 4.2.3. Intestinal Pseudo-Obstruction

Intestinal pseudo-obstruction manifests as bowel dilatation without anatomical obstruction [39]. Abdominal distention is a hallmark feature of intestinal pseudo-obstruction, and other common symptoms include nausea, vomiting, constipation, and early satiety. Intestinal pseudo-obstruction comprises acute and chronic states [39].

Acute pseudo-obstruction, also known as Ogilvie syndrome, most commonly affects the large intestine and typically follows surgery or critical illness. While its pathophysiology remains unclear, it is hypothesized to be related to a poorly regulated autonomic nervous system. Chronic pseudo-obstruction is rarer than acute pseudo-obstruction and occurs secondary to neuropathies, myopathies, or abnormalities of the interstitial cells of Cajal [39].

## 5. Management and Treatment

It is important to recall that constipation management is not a one-size-fits-all approach, as some therapeutic strategies may be more effective than others depending on the underlying pathophysiology [21,37,40]. Constipation management in all inpatient cases should begin with nonpharmacologic approaches, such as ensuring that patients are adequately hydrated, correcting electrolyte imbalances, ensuring adequate fiber intake, and encouraging ambulation (Figure 3) [2,7]. In the case of fecal impaction, manual disimpaction, which should precede the use of an oral laxative regimen, may be required [33].

### 5.1. Laxatives and Stool Softeners

Pharmacologic treatment strategies may be necessary to adequately treat constipation, especially in patients with OIC [6,21,35]. Laxatives are a common first-line treatment, largely due to widespread availability and low cost (Figure 3) [35]. Accordingly, the American Gastroenterological Association’s Consensus Guideline for the management of OIC strongly recommends a trial of laxatives as an initial intervention [34]. Traditional laxatives include osmotic agents, stimulants, stool softeners, and lubricants [34]. Osmotic agents increase stool water, and accordingly improve stool consistency. Magnesium preparations are a type of osmotic laxative with rapid, potent effects and are often used as colon purgatives prior to colonoscopy or surgery. Saline laxatives and polyethylene glycol are preferred over the nonabsorbed sugars and sugar-alcohols (e.g., lactulose and sorbitol); the latter have the potential to ferment and be metabolized by intestinal flora, leading to undesirable gas and bloating [35]. Stimulants activate sensory nerve endings to promote colonic motility and reduce colonic water absorption. Stool softeners work by softening feces via the penetration of water and lipids. Lubricants work by lubricating the lining of the gut to enable defecation [34]. While generally safe, the use of laxatives is associated with gastrointestinal adverse effects, including bloating, diarrhea, and abdominal fullness [35]. Clinical trial evidence supporting the benefit of laxatives in hospitalized patients is limited. Existing trials were of short duration and used endpoints that are difficult to translate to clinical practice in the acute care setting, such as stool consistency and bowel movement frequency [41].

### 5.2. Enemas

Enemas are often used to treat constipation in the inpatient setting, partly because the effects of laxatives can be slow and unpredictable. There is limited clinical evidence of the efficacy of enemas, and they may pose safety concerns, including electrolyte disturbances, rectum mucosal damage, and megacolon [2].

### 5.3. Intestinal Secretagogues and Prokinetics

Intestinal secretagogues act on enteric chloride channels or guanylate cyclase receptors to establish an electrolyte gradient in the bowel lumen, resulting in enhanced water secretion into the intestinal lumen [34,35]. The most common adverse event with these agents is diarrhea. Selective 5-hydroxytryptamine (5-HT) agonists, including prucalopride, act through 5-HT4 receptors in the gastrointestinal tract, facilitating increased colonic motility and transit [34,35]. The acetylcholinesterase inhibitor neostigmine may be considered in patients with severe functional colonic pseudo-obstruction to stimulate gut motility [7].

### 5.4. Peripherally Acting µ-Opioid Receptor Antagonists

Although first-line treatment with laxatives is generally regarded to be safe and cost-effective, they do not target the underlying pathophysiology of OIC (i.e., opioid binding of opioids to gastrointestinal μ-opioid receptors) [21]. Unlike laxatives, peripherally acting µ-opioid receptor antagonists (PAMORAs) preferentially block bowel µ-opioid receptors, thereby targeting the primary opioid-related mechanism underlying constipation [34]. However, the use of PAMORAs in clinical practice may be limited due to the higher cost of these drugs [42]. Currently, naldemedine, naloxegol, and methylnaltrexone are indicated for OIC; alvimopan is indicated specifically for postoperative ileus [34,35,37]. Abdominal pain, diarrhea, and nausea are the most common adverse effects of PAMORAs [34,43,44,45]. PAMORAs are contraindicated in cases of known or suspected gastrointestinal obstruction [43,44,45].

Naloxegol, a PEGylated derivative of naloxone, is approved for the treatment of OIC in adult patients with chronic noncancer pain, including patients with chronic pain related to prior cancer or its treatment who do not require frequent (weekly) opioid dosage escalation [34,43]. This medication can also be crushed for enteral tube administration or for patients with swallowing difficulties [43].

Methylnaltrexone is available as a tablet or subcutaneous injection for the treatment of OIC in adults with chronic noncancer pain and as an injection for adults with advanced illness or pain caused by active cancer [44]. Subcutaneous formulations may be particularly useful in hospitalized patients who may not be able to take orally administered medicines and require management of complex symptoms with frequent medication administration [46]. The oral formulation has the benefit of simplifying administration without compromising opioid analgesia [47].

Naldemedine is an oral PAMORA approved for OIC treatment in adult patients with chronic noncancer pain, including patients with chronic pain related to prior cancer or its treatment who do not require frequent (e.g., weekly) opioid dosage escalation [45]. However, given its metabolism, naldemedine use should be avoided in combination with medications that inhibit CYP3A4.

### 5.5. Surgical Interventions

If other therapies have failed, surgical interventions may be appropriate for some patients, depending on the dominant underlying pathophysiology and contraindications. Surgical procedures are associated with potential risks and morbidity, so they should be a last-resort therapy. Surgical options have previously been reviewed [41,48] and are only briefly summarized here. Patients with refractory slow-transit constipation or chronic pseudo-obstruction may benefit from colon resection [39,41,48]. Colectomy is estimated to improve constipation and quality of life in over half of patients [48,49]; however, complications, such as small bowel obstruction, diarrhea, incontinence, and abdominal pain, can occur, leading to rehospitalization and potentially reoperation [41,49]. In patients with intractable constipation, stoma creation may be considered. Up to 70% of patients are satisfied with stoma interventions, although complications are common [41]. Loop ileostomy is a potentially reversible alternative that clinicians can attempt before colectomy or stoma creation [41].

## 6. Conclusions

The effective recognition and management of constipation requires a systemic approach and relies on taking a thorough patient history, the use of validated assessments, physical examination, and, in some cases, radiographic imaging studies. Treatment of constipation in the hospitalized patient requires a tailored approach, as some therapeutic strategies may be more effective than others, depending on the pathophysiology of the constipation. The need for individualized approaches to constipation management is especially true in patients with OIC, where traditional approaches, such as nonpharmacologic therapies and laxatives, may not be sufficient to address symptoms. In such cases, PAMORAs may be more effective and appropriate.

## Data Availability

Not applicable.
